# A nomogram for predicting the risk of *Clostridioides difficile* infection in children with ulcerative colitis: development and validation

**DOI:** 10.3389/fped.2025.1641220

**Published:** 2025-10-09

**Authors:** Ziying Li, Fumin Xue, Jing Zhang, Zhidan Yu, Xiaoqin Li, Yuesheng Wang

**Affiliations:** ^1^ Department of Gastroenterology, Children's Hospital Affiliated to Zhengzhou University, Zhengzhou, Henan, China; ^2^ Zhengzhou University, Zhengzhou, Henan, China; ^3^ Department of Clinical Nutrition, Children's Hospital Affiliated to Zhengzhou University, Zhengzhou, Henan, China; ^4^ Zhengzhou Key Laboratory of Children's Digestive Diseases, Children's Hospital Affiliated to Zhengzhou University, Zhengzhou, Henan, China

**Keywords:** ulcerative colitis, *Clostridioides difficile*, children, nomograms, risk factors

## Abstract

**Introduction:**

This study aimed to develop a dynamic nomogram model to predict the risk of *Clostridioides difficile* infection (CDI) in children with ulcerative colitis (UC).

**Methods:**

This was a retrospective study that clinical data from pediatric diagnosis and treatment with UC at Zhengzhou University Children's Hospital between January 2018 and December 2024 were retrospectively reviewed. Patients were classified into CDI (*n* = 35) and non-CDI (*n* = 86) groups based on the presence or absence of CDI. Predictor variables were selected using least absolute shrinkage and selection operator (LASSO) regression and subsequently entered into a multivariate logistic regression model. Nomograms were then constructed based on the final logistic regression analysis. The model's performance and clinical utility were assessed using receiver operating characteristic (ROC) curves, calibration plots, and decision curve analysis (DCA). Internal validation was performed using 1,000 bootstrap resamples.

**Results:**

A total of 121 children were included in the study. Based on LASSO and multivariate logistic regression analysis of 24 candidate variables, five independent risk factors for CDI in children with UC were identified: Pediatric Ulcerative Colitis Activity Index (PUCAI), erythrocyte sedimentation rate (ESR), vitamin D (Vit D), fecal calprotectin (FC), and antibiotic use exceeding seven days (all *p* < 0.05). The nomograms constructed with the above variables demonstrated excellent discriminative ability (C-index = 0.964, 95% CI: 0.932–0.997). The Hosmer-Lemeshow test (χ^2^ = 12.529, *p* = 0.129*)* and bootstrap validation revealed good concordance between the predicted probabilities and actual outcomes. Decision curve analysis (DCA) indicated significant net clinical benefit, and the model maintained robust consistency across relevant clinical subgroups.

**Conclusions:**

PUCAI, ESR, Vit D, FC, and use of antibiotic use exceeding seven days were the five independent risk factors for CDI in children with UC. The resulting nomogram may support clinicians in early diagnosis and timely adjustment of therapeutic strategies.

## Introduction

Ulcerative colitis (UC) is a chronic, relapsing-remitting inflammatory disease characterized by mucosal diseases confined to the colon, with approximately 15%–20% of cases presenting during childhood or adolescence (1). Its pathogenesis involves multifactorial mechanisms, including genetic susceptibility, environmental exposures (e.g., infections, diet, and lifestyle), gut microbiota alterations, and immune dysregulation. Compared to adult-onset UC, children with UC typically presents with abrupt onset, rapid progression, extensive mucosal involvement, and an unpredictable course marked by alternating remission and acute exacerbations. Clinical management is often complicated by frequent hospitalizations, a high rate of corticosteroid resistance, and a colectomy rate reaching 5%–6% within one year of diagnosis (2, 3). Despite the efficacy of long-term immunosuppressive therapies - such as 5-aminosalicylic acid (5-ASA), systemic and topical corticosteroids, immunomodulators, and biologics - in inducing and maintaining remission, these treatments increase susceptibility to opportunistic infections, suboptimal mucosal healing, and secondary loss of therapeutic response. Among opportunistic infections, *Clostridioides difficile* infection (CDI) and cytomegalovirus (CMV) represent significant contributors to disease exacerbation in children with UC.

CDI poses substantial public health challenges due to its high incidence, recurrence, and associated mortality. According to Centers for Disease Control and Prevention (CDC) surveillance data, community-associated CDI accounted for 75% of pediatric cases in 2019, with an incidence reaching 25.8 per 100,000 children (4). CDI is a significant health concern with consequences for pediatric's growth and overall morbidity. Individuals with inflammatory bowel disease (IBD), including both adults and children, exhibit increased susceptibility, with CDI prevalence rates of 13% and 6.9%, respectively (5, 6). Although *Clostridioides difficile* (CD) is a non-invasive, spore-forming, obligate anaerobic *Gram-positive bacillus*, it produces enterotoxins (toxins A and B) and tissue-degrading enzymes (e.g., collagenase, hyaluronidase, chondroitin sulfate lyase) that compromise epithelial integrity by disrupting tight junctions and the actin cytoskeleton. This cascade leads to fluid secretion, localized inflammation, and a spectrum of clinical manifestations ranging from mild diarrhea to life-threatening complications such as pseudomembranous colitis, toxic megacolon, intestinal perforation, systemic inflammatory response syndrome (SIRS), sepsis, and death (7).

The treatment strategy for CDI depend on the severity of the infection, the presence of recurrence, and individual patient factors. The main antibiotic options include metronidazole, vancomycin, or fidaxomicin. For initial CDI episodes, it is recommended to first discontinue the suspected inciting antibiotics. Vancomycin or metronidazole are typically used as first-line agents, with fidaxomicin considered in select cases (8). In cases of recurrent CDI, distinct therapeutic approaches are indicated: (1) For patients whose initial episode was treated with vancomycin or metronidazole, fidaxomicin is recommended for the first recurrence; (2) For those initially treated with fidaxomicin, adjunctive bezlotoxumab therapy should be combined with standard oral antibiotic regimens (vancomycin or fidaxomicin). Multiple recurrent CDI cases warrant fecal microbiota transplantation (FMT) or bezlotoxumab supplementation following standard antibiotic pretreatment (7). FMT can regulate the gut microbiota and has been approved by the FDA for the treatment of recurrent CDI. However, the results of FMT in the treatment of UC are inconsistent and its efficacy is questionable. Large-scale randomized controlled trials have not been conducted for verification and inclusion in the pediatric population (9). Therefore, this study excluded pediatric treated with FMT.

Due to overlapping clinical manifestations - such as fever, abdominal pain, and diarrhea - differentiating between a acute children with UC and concurrent CDI presents a diagnostic challenge. Several diagnostic modalities for CDI are currently employed, including enzyme immunoassays (EIA) for toxins A and B, glutamate dehydrogenase (GDH) detection, nucleic acid amplification tests (NAAT), toxin-producing culture, and next-generation sequencing (NGS) (10). However, these techniques differ in sensitivity and specificity. Given these limitations, the development of a simple, rapid, and noninvasive predictive tool is critical for children with UC at high risk of CDI. This study constructed nomograms model to predict CDI risk in children with UC. The model offers a clinically practical approach to facilitate early diagnosis and prompt initiation of standardized treatment, improving clinical outcomes in children with UC combined with CDI.

## Methods

### Study population

This retrospective, single-center clinical study was conducted at the Affiliated Children's Hospital of Zhengzhou University. Children admitted with UC between January 2018 and December 2024 were identified through the electronic medical records system using the search terms “ulcerative colitis” and/or “*Clostridioides difficile* infection” in accordance with the 2019 *Expert Consensus on the Diagnosis and Treatment of Inflammatory Bowel Disease in Children* (11). Inclusion criteria were as follows: (1) a confirmed UC diagnosis (for at least 3 months) (12); (2) age between 0 and 18 years; and (3) performed and documented CD testing (CDI or non-CDI). Exclusion criteria included: (1) severe cardiac, pulmonary, hepatic, or renal dysfunction; (2) prior use of metronidazole or vancomycin within one month before admission; (3) vitamin D (Vit D) supplementation within the previous six months (to avoid potential confounding effects of exogenous Vit D supplementation on disease progression in children with UC combined with CDI); (4) history of fecal microbiota transplantation; (5) and incomplete clinical records lacking any of the 24 potential predictor variables. Based on these criteria, 35 children were ultimately enrolled. The remaining patients did not meet the inclusion criterion of documented CDI.

The diagnosis of CDI was based on the 2017 *Clinical Practice Guidelines for CDI in Adults and Children* (13) from the Infectious Diseases Society of America (IDSA) and Society for Healthcare Epidemiology of America (SHEA), which define CDI as the presence of gastrointestinal symptoms (diarrhea, increased frequency of bowel movements, bloody stools, intestinal spasms, and/or urgency) combined with one of the following criteria: (1) positive for CD GDH and positive for CD toxin A or B by enzyme-linked immunosorbent assay; (2) positive for CD GDH, negative for CD toxin A and B by EIA, and positive for CD by real-time quantitative polymerase chain reaction (PCR); and/or (3) colonoscopy or histopathology showing pseudomembranous enteritis.

The study protocol was approved by the Ethics Committee of the Affiliated Children's Hospital of Zhengzhou University (approval number: 2024-079-002), and all data were anonymized prior to analysis.

### Disease assessment standards

Disease activity was stratified into active and remission phases. Within the active phase, severity was further subcategorized as mild, moderate, or severe based on clinical criteria. The Pediatric Ulcerative Colitis Activity Index (PUCAI) was employed to assess disease activity (14): remission (PUCAI < 10), mild activity (10–34), moderate activity (35–64), and severe activity (>65). In this study, disease activity in UC is categorized into two main groups: primary relapse and chronic relapsing UC. Primary relapse refers to patients who experience a disease flare after initially achieving remission following diagnosis based on as sustained clinical remission (PUCAI < 10). Chronic relapsing UC refers to patients who experience ≥1 disease flare per year, which could be confirmed by endoscopy. These relapses are further classified based on frequency: infrequent (one episode or fewer per year), frequent (two or more episodes per year), or continuous (persistent UC symptoms without achieving remission). Additionally, an early relapse is defined as a recurrence of symptoms occurring within less than three months after remission was achieved through prior treatment (15).

Disease extent was classified according to the Paris classification system (16). Extraintestinal manifestations included ocular involvement (e.g., iritis, scleritis, uveitis), hepatobiliary disorders (e.g., fatty liver, primary sclerosing cholangitis, cholelithiasis), dermatologic and mucosal findings (e.g., oral ulcers, erythema nodosum, pyoderma gangrenosum), musculoskeletal complications (e.g., peripheral arthritis, spondyloarthritis), and thromboembolic events (17). Additional complications comprised intraepithelial neoplasia of the intestinal mucosa, bowel perforation, lower gastrointestinal hemorrhage, toxic megacolon, and malignancy.

### Data collection

Clinical data were retrieved from the institution's electronic medical database in accordance with principles of data accessibility and clinical relevance. Extracted variables included: (1) demographic and clinical characteristics, including age (years), sex, body mass index (kg/m^2^), family history, presence of extraintestinal manifestations, upper gastrointestinal involvement, comorbidities, clinical relapse type, extent of colonic diseases, PUCAI, and antibiotic use exceeding seven days (days); (2) laboratory parameters, including white blood cell count (*10^9^/L), hemoglobin (g/L), platelet count (mg/L), albumin (g/L), erythrocyte sedimentation rate (mm/h), procalcitonin (ng/ml), interleukin-6 (pg/ml), tumor necrosis factor-α (pg/ml), immunoglobulin A (g/L), Vitamin D (nmol/L), CD4^+^/CD8^+^ T-cell ratio (%), fecal calprotectin (ug/g), CMV IgM status, and CDI results (CD toxin genes were detected in children with UC fecal samples by real-time quantitative PCR). The PUCAI and laboratory parameters were selected for the period between admission and CDI testing (before the child developed symptoms that required CDI testing).

### Statistical analysis

Statistical analyses were performed using SPSS version 26.0. For continuous variables with normal distribution, data were expressed as mean ± standard deviation (χ¯ ± s) and compared using the independent samples *t*-test. Non-normally distributed continuous variables were reported as median and interquartile range (P_25_, P_75_) and analyzed using the Mann–Whitney *U*-test. Categorical variables were presented as frequencies and percentages (%) and compared using the χ^2^ test.

To enhance the generalizability of the predictive model, the least absolute shrinkage and selection operator (LASSO) regression was applied to identify key features associated with children with UC combined with CDI. Variables selected by LASSO regression were subsequently entered into a multivariate logistic regression model. Statistically significant predictors were incorporated into the construction of a nomogram, with corresponding odds ratios (ORs), 95% confidence intervals (CIs), and *P*-values reported. Internal validation was conducted using a 1,000-sample bootstrap resampling technique. Model performance, including discrimination, calibration and clinical utility, was assessed through receiver operating characteristic (ROC) curves, calibration plots, and decision curve analysis (DCA). Model construction and validation were performed using R software (version 4.2.4). A two-tailed *P* < 0.05 was considered statistically significant.

## Results

### Screening for optimal predictive markers of children with UC combined with CDI

**Figure 1:**
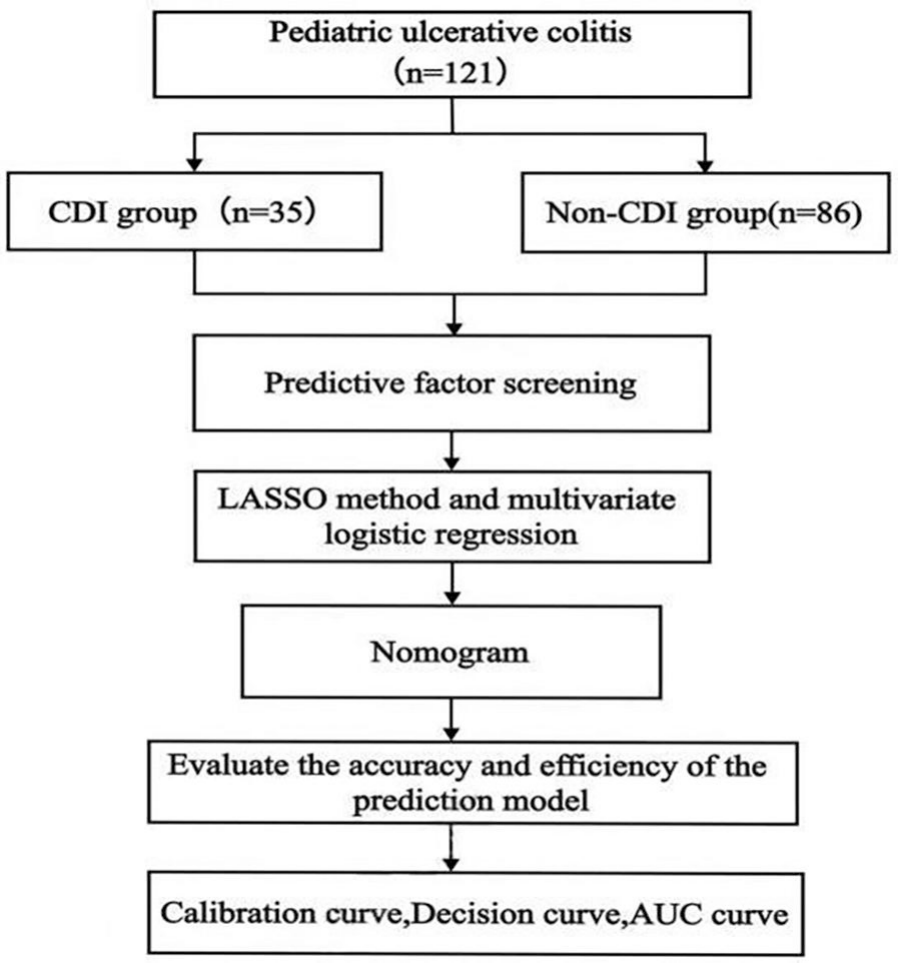
The flow chart illustrating the research methodology.

**Figure 2:**
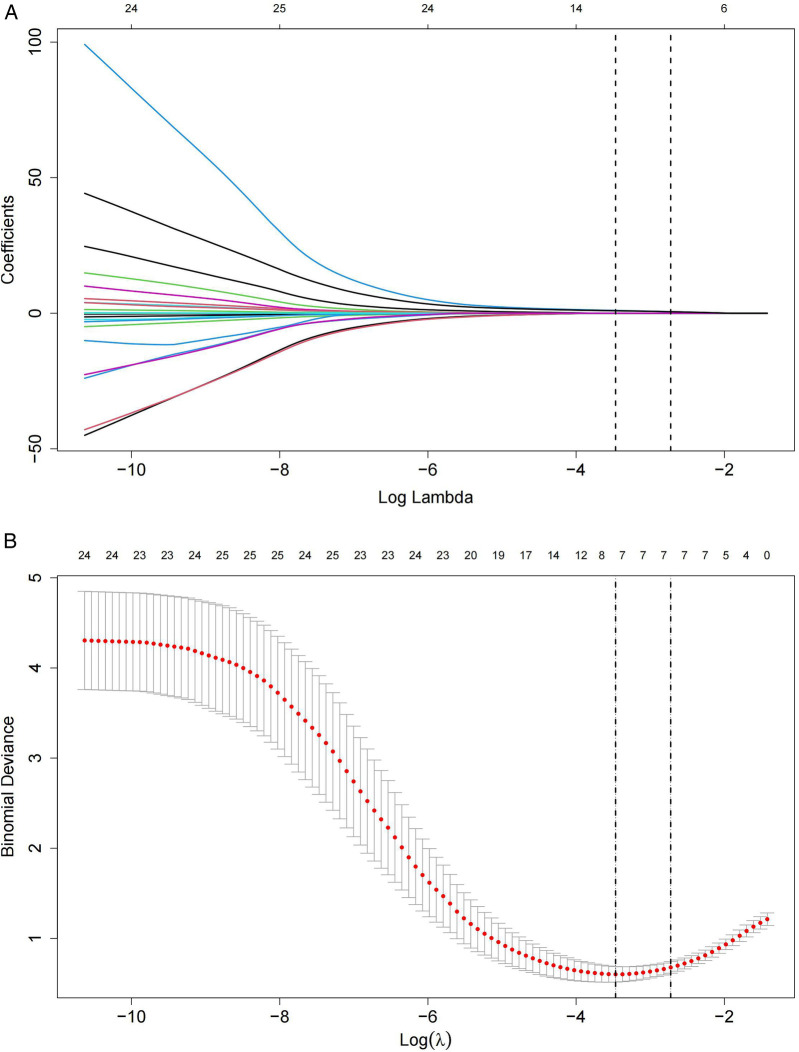
Lasso regression model of children with UC combined with CDI. **(A)** Lasso coefficient profile plot for 24 feature predictors, with variable values marked at the top of the plot. **(B)** The Lasso regression model's parameters were selected using five-fold cross-validation and the minimum criteria. Seven non-zero coefficients were identified by drawing two vertical lines at the optimal point of the minimum mean squared error (left) and one standard error above the minimum mean squared error (right).

All eligible patients were categorized into two groups: CDI (*n* = 35) and non-CDI (*n* = 86) based on the presence or absence of combined with CDI (Figure 1). The median time interval from children with UC diagnosis to the collection of disease activity scores and laboratory parameters was 1 (0.5–2) days. The median time interval from CDI diagnosis to completion of the aforementioned assessments was 3 (2.5–4) days. The baseline characteristics of the patients are shown in Table 1. Based on non-zero coefficients identified through LASSO logistic regression, seven candidate predictors were selected from the initial pool of 24 variables (Figures 2A,B), including PCUAI, Hb, ESR, Vit D, FC, and antibiotic use exceeding seven days. These variables were subsequently entered as independent predictors in a binary logistic regression model, with the presence of CDI in children with UC as the dependent variable. Multivariate logistic regression analysis identified PUCAI, ESR, Vit D, FC, and prolonged antibiotic use as independent risk factors for CDI in children with UC (Table 2).

**Table 1:** Comparison of clinical characteristics between UC Patients with CDI or non-CDI groups.

Variables	CDI group (*n* = 35)	non-CDI group (*n* = 86)	*P*
Age, median (IQR)	10.00 (7.12–11.00)	5.30 (2.64–9.00)	<0.001
BMI, median (IQR)	15.04 (14.14–16.84)	15.89 (14.58–18.14)	0.073
Genders			
Male	20 (57.14%)	51 (59.30%)	0.988
Female	15 (42.86%)	35 (40.70%)	
Family history			
No	26 (74.29%)	67 (77.91%)	0.849
Yes	9 (25.71%)	19 (22.09%)	
Extraintestinal manifestations			
No	14 (40.00%)	62 (72.09%)	0.002
Yes	21 (60.00%)	24 (27.91%)	
Involving UGT			
No	26 (74.29%)	75 (87.21%)	0.143
Yes	9 (25.71%)	11 (12.79%)	
Complication			
No	25 (71.43%)	65 (75.58%)	0.807
Yes	10 (28.57%)	21 (24.42%)	
Clinical type			
Chronic relapse	10 (28.57%)	51 (59.30%)	0.004
Primary relapse	25 (71.43%)	35 (40.70%)	
Location of disease			
E1 + E2	12 (34.29%)	62 (72.09%)	<0.001
E3 + E4	23 (65.71%)	24 (27.91%)	
PUCAI			
Mild active phase	8 (22.86%)	53 (61.63%)	<0.001
Moderate to severe active phase	27 (77.14%)	33 (38.37%)	
WBC, median (IQR)	8.24 (7.08–11.30)	8.43 (6.07–10.90)	0.591
Hb, mean (SD)	102.49 (17.92)	121.92 (15.91)	<0.001
PLT, median (IQR)	378.00 (280.50–517.00)	307.50 (255.25–393.00)	0.018
ALB, median (IQR)	38.40 (33.20–44.90)	43.05 (39.70–46.68)	0.016
ESR, median (IQR)	26.00 (10.50–47.00)	10.00 (6.00–14.75)	<0.001
PCT, median (IQR)	0.07 (0.04–0.08)	0.04 (0.02–0.07)	0.014
IL-6, median (IQR)	11.36 (4.73–22.59)	5.46 (2.87–14.47)	0.021
TNF-α, median (IQR)	22.10 (11.25–65.50)	24.35 (12.35–69.08)	0.801
IgA, median (IQR)	1.50 (0.88–2.38)	1.04 (0.62–1.49)	0.004
VitD, median (IQR)	14.45 (9.53–18.06)	44.96 (29.33–66.49)	<0.001
Lymphocyte subset analysis			
CD4+, mean (SD)	38.73 ± 9.18	35.93 ± 9.10	0.131
CD8+, median (IQR)	27.74 (22.32–33.07)	26.41 (22.00–30.90)	0.313
FC, median (IQR)	114.70 (60.40–497.95)	28.85 (15.00–40.51)	<0.001
CMV IgM:			
Positive	26 (74.29%)	76 (88.37%)	0.098
Negative	9 (25.71%)	10 (11.63%)	
Antibiotic use exceeding seven days			
No	9 (25.71%)	59 (68.60%)	<0.001
Yes	26 (74.29%)	27 (31.40%)	

BMI, body mass index; UGT, upper gastrointestinal tract; PUCAI, pediatric ulcerative colitis activity index; WBC, white blood cell; Hb, hemoglobin; PLT, platelet; ALB, albumin; ESR, erythrocyte sedimentation rate; PCT, procalcitonin; IL-6, interleukin-6; TNF-α, tumor necrosis factor-α; IgA, immunoglobulin A; Vit D, vitamin D; CD4^+^/CD8^+^, cluster of differentiation 4^+^/cluster of differentiation 8^+^; FC, fecal calprotectin.

**Table 2:** Multivariable logistic regression analysis of children with UC combined with CDI.

Variables	OR	95% CI	*P*
Age	1.088	0.911–1.311	0.351
PUCAI	8.354	1.858–52.47	0.01
Hb	0.968	0.914–1.018	0.224
ESR	1.048	1.009–1.101	0.035
Vit D	0.946	0.906–0.977	0.003
FC	1.004	1.001–1.009	0.026
Antibiotic use exceeding seven days	5.205	1.135–28.73	0.04

### Development of a prediction model

**Figure 3:**
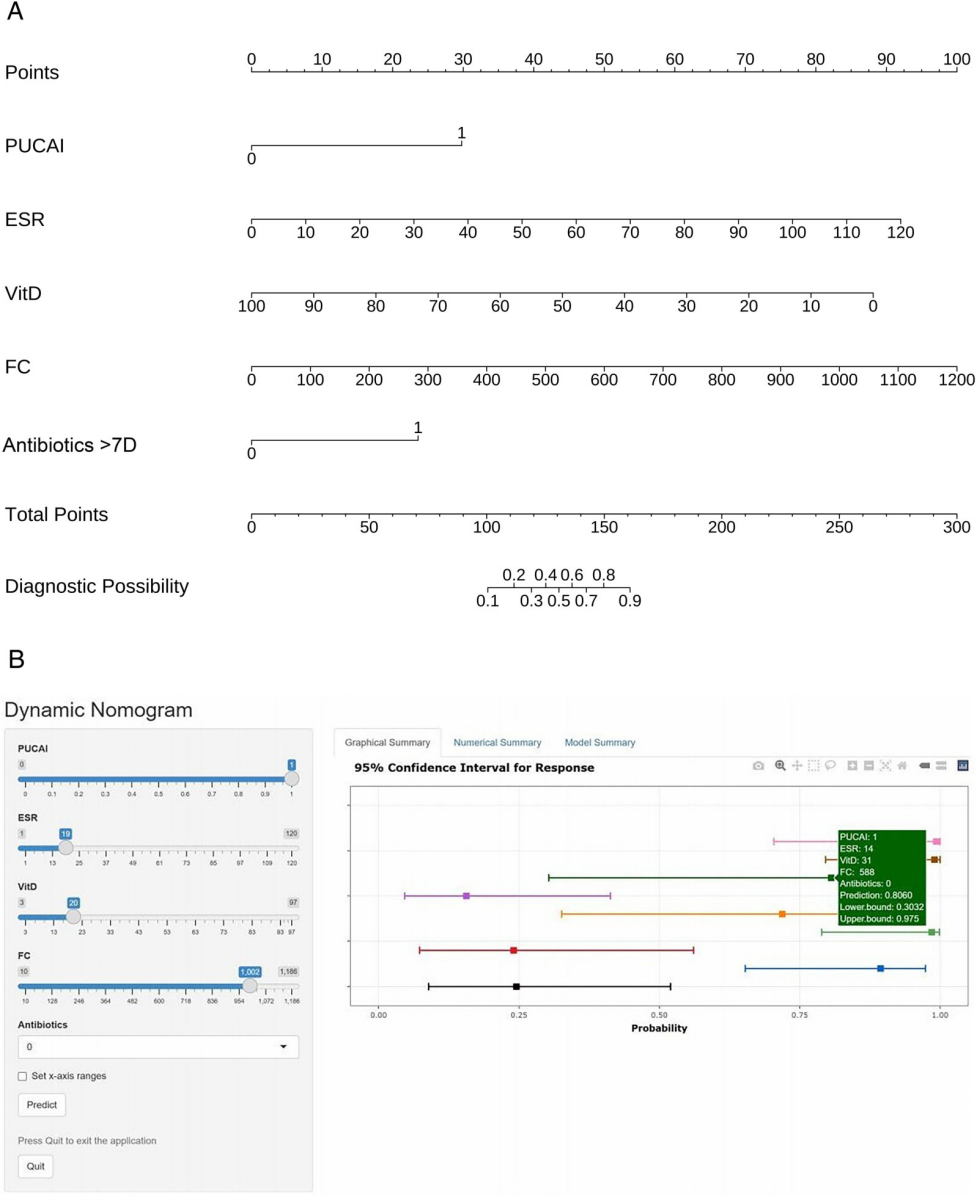
Construction of static-dynamic nomogram prediction models for children with UC combined with CDI. **(A)** Static nomogram for children with UC combined with CDI. **(B)** Online dynamic nomogram for children with UC combined with CDI. (https://plotsite.shinyapps.io/nomograme/).

Two forms of nomograms was developed using five independent predictors that were statistically significant in the multivariate logistic regression analysis (Figure 3). In the static nomogram, each variable contributed to the calculation of its respective score, and the overall score was obtained by summing these individual scores. The risk probability of CDI in children with UC was determined by mapping the overall score to the corresponding value on the risk axis. In this model, higher total scores correlate with an increased likelihood of CDI occurrence in children with UC. The dynamic nomogram enables real-time predictive utility by displaying the risk of CDI and its 95% CI upon entering five variables per patient.

To determine the score cut-off values for low, medium, and high wind directions, the maximum Youden's Index (YI) in the ROC curve was used to select the row with the largest YI from the data, and the corresponding threshold was found. Based on clinical practicability and data distribution, three-level stratification was carried out (Table 3). High risk: select the point with the largest YI; Medium risk: the lower limit is set at sensitivity = 100% and specificity = 50%, balancing the rate of missed diagnosis and false diagnosis; the upper limit is the YI maximum point; Low risk: the sensitivity is close to 100%, which can basically rule out the risk of CDI.

**Table 3:** Risk stratification for children with UC combined with CDI.

Level of risk	Total scores	Clinical recommendations
Low risk	≤74 scores	No intervention needed, routine follow-up
Moderate risk	74–118 scores	Test for CDI
High risk	≥118 scores	Start empiric anti-CDI therapy with concurrent testing

### Predictive model performance and validation

**Figure 4:**
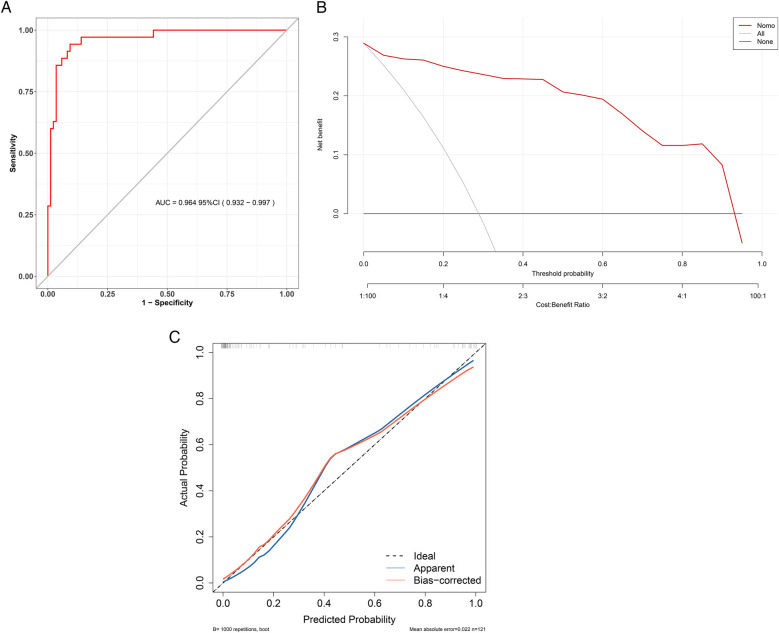
Performance evaluation of a children with UC combined with CDI prediction model. **(A)** ROC curve for children with UC combined with CDI. The *y*-axis represents the sensitivity, while the *x*-axis represents 1-specificity. The ROC curve would align with the gray diagonal if the model's performance were equivalent to random guessing. The red curve illustrates the model's sensitivity and 1-specificity at various thresholds, with greater proximity to the upper left corner indicating higher model accuracy. **(B)** DCA for children with UC combined with CDI. In the DCA, the red line represents the risk model for children with UC combined with CDI. The thin line corresponds to the strategy of assuming all children have children with UC combined with CDI, while the thick line represents the strategy of assuming none have children with UC combined with CDI. Model validation. **(C)** The *x*-axis represents the predicted risk of children with UC combined with CDI, while the *y*-axis represents the actual diagnosis of children with UC combined with CDI. The red solid line indicates the performance of the nomogram, and the black dotted line represents the ideal calibration line. A closer alignment of the red line to the black dotted line signifies better model calibration.

**Figure 5:**
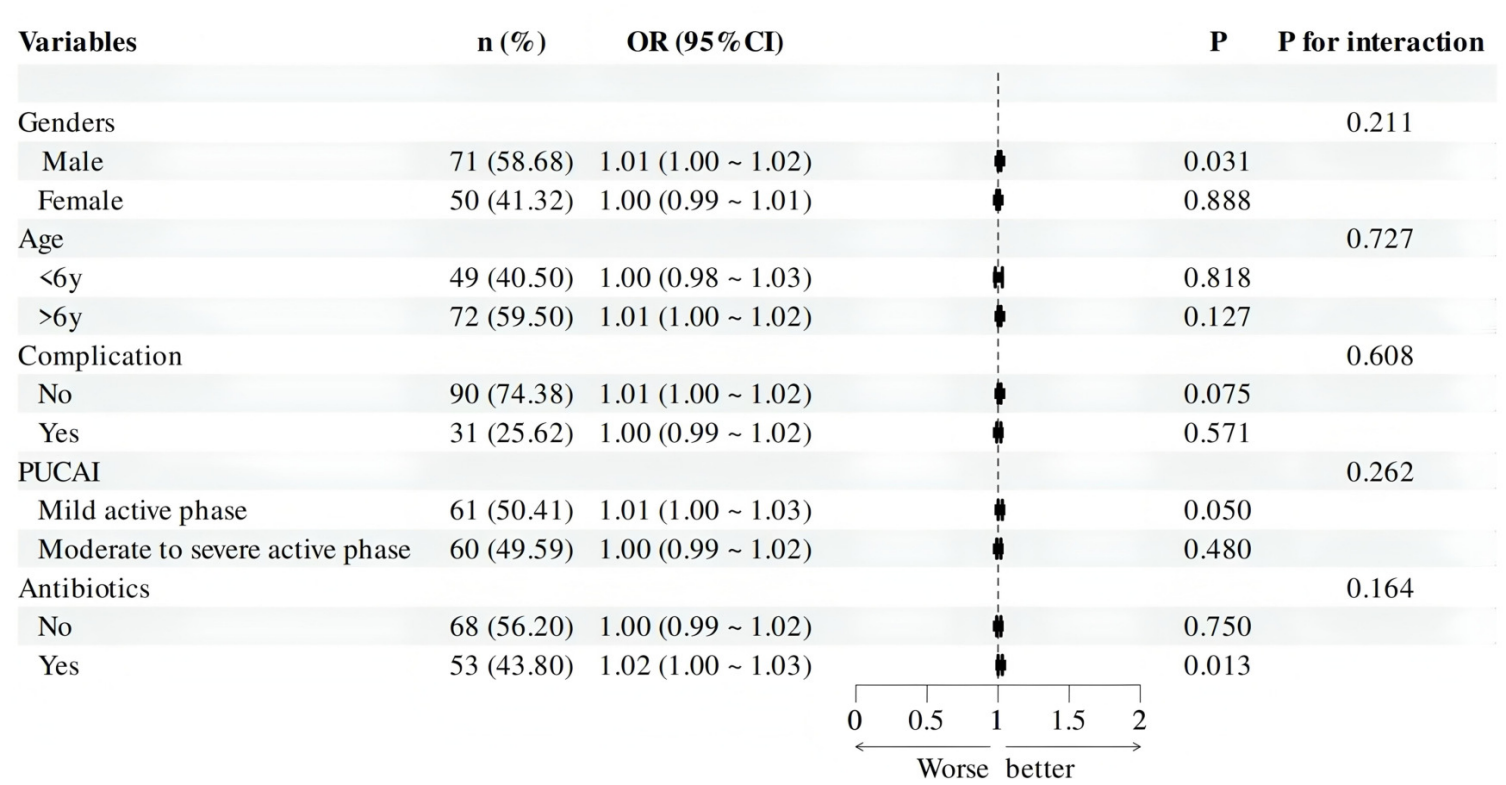
Forest plot of subgroup analysis for children with UC combined with CDI.

The Hosmer-Lemeshow test was used to evaluate the model fit. The results showed that χ^2^ = 12.529, *p* = 0.129, indicating that the model does not have poor goodness of fit. The area under the ROC curve (AUC) reflects the model's ability to distinguish between different patient categories (Figure 4A). The C-index of this model was 0.964 (95% CI: 0.932–0.997), indicating that it can predict children with UC combined with CDI with a 96.4% confidence level. The sensitivity, specificity, PPV, and NPV were 80.4%, 90.6%, 80.4%, and 97.5%, respectively. The clinical applicability of the model was evaluated using DCA (Figure 4B). The results revealed that, across a broad range of threshold probabilities, the model's net benefit consistently exceeded that of the two extreme strategies (including all children with UC combined with CDI or none of them combining CDI), highlighting its potential clinical utility. Internal validation was completed using 1,000 Bootstrap analyses, and the results showed that the model has the ability to distinguish children with UC combined with CDI, with an average absolute error of only 0.022 (Figure 4C). Further evaluation of the model's performance in clinically relevant subgroups was conducted. The subgroup analysis of the forest plot revealed that the predictive efficacy of the model in terms of demographic and clinical characteristics was consistent (*p* > 0.05), indicating that the model can accurately and simply predict the risk of CDI in patients with UC (Figure 5).

## Discussion

CDI is a common opportunistic pathogen affecting both adults and pediatric patients, particularly in those with IBD. Adult studies have demonstrated that patients with UC are more susceptible to CDI (18). This predisposition may be attributed to the pathological characteristics of UC, which typically presents as continuous inflammatory lesions affecting the colorectum mucosa. Concurrent CDI in UC patients is associated with prolonged hospitalization, increased frequency of disease relapse, suboptimal therapeutic response to medications, and elevated risk of colectomy. Consequently, early clinical identification of such patients has emerged as a critical issue in IBD management. To date, there remains a scarcity of research on risk factors for CDI in children with UC populations, and no standardized diagnostic protocol for CDI detection has been established. To facilitate real-time clinical decision-making, this study developed an intuitive dynamic nomogram prediction tool utilizing readily accessible clinical parameters. This instrument enables risk stratification and prediction of CDI infection, thereby providing a valuable resource for proactive clinical intervention.

Based on fecal and blood biomarkers, feature selection was performed using Lasso regression, ultimately identifying five variables for inclusion in the risk prediction model for children with UC complicated by CDI. To guide clinical practice, the optimal cut-off value maximizing the YI was applied for risk stratification. For low-risk patients, regular follow-up is recommended; intermediate-risk patients should undergo CDI testing; and high-risk patients should receive empirical anti-CDI therapy concurrently with diagnostic testing. Internal validation demonstrated excellent performance with a mean absolute error of 0.022, indicating that the model provides stable and reliable prediction of CDI risk in children with UC patients.

To evaluate the predictive accuracy of the model, we conducted subgroup analyses across clinically relevant categories (gender, age, comorbidities, disease activity, and treatment history). Forest plot results demonstrated consistent predictive performance across demographic and clinical characteristics. A recent systematic review and meta-analysis on CDI in IBD patients identified age and male gender as significant risk factors for CDI (19). The discrepancy between our findings and previous reports may be attributed to population differences, as the present study exclusively enrolled pediatric patients under 18 years of age.

Diagnostic and therapeutic decisions for children with UC rely on disease activity, as measured by PUCAI, a scoring system used to assess disease severity in children with UC. Findings from this study reveal that the PUCAI demonstrates significant predictive utility for clinical outcomes in adult patients with acute severe colitis (20). Strong suspicion of CDI is warranted in children with elevated PUCAI. Most patients with IBD and CDI exhibit moderate to severe PUCAI (33% and 66%, respectively) (21). The results of the multivariate regression analysis in this study showed that moderate to severe children with UC, as indicated by PUCAI, was identified as an independent risk factor for concurrent CDI. Subgroup analysis conducted by Fang et al. similarly demonstrated a significant association between disease activity and CDI (22). Active disease stages, characterized by impaired mucus layer formation and compromised epithelial barrier function, may increase the susceptibility of children with UC to pathogenic colonization and invasion. Thus, PUCAI was incorporated into a nomogram model children with UC combined with CDI. A bidirectional relationship exists between CDI and changes in disease activity: while elevated IBD disease activity may increase susceptibility to CDI, the infection itself can conversely exacerbate IBD severity (23). Some studies suggest this observed association may be attributable to symptomatic overlap and disproportionate CDI testing in active disease states. Further investigation is required to elucidate this potential detection bias and establish causal mechanisms (22).

ESR and FC are both indicators reflecting the inflammatory activity of children with UC. In this study, children with UC combined with CDI exhibited elevated ESR and FC levels. At the same time, there are also research results showing that FC levels correlate positively with PUCAI, ESR, PLT, and CRP in children with UC (24). ESR, cost-effective and clinically practical biomarker for assessing inflammatory activity, reflects the rate of erythrocyte sedimentation in plasma, which is accelerated by elevated concentrations of globulin, fibrinogen, and complement. In IBD diagnosis, ESR demonstrates a sensitivity of 66% and specificity of 84% (25). It correlates with endoscopic inflammatory activity in children with UC and serves as a laboratory marker to predict disease severity. Changes in the gut microbiota and metabolites can trigger immune responses in epithelial cells, resulting in systemic inflammation. Wan et al. reported that certain metabolites elevated in UC patients with CDI, such as putrescine, maltose, 4-hydroxybenzoic acid, 4-hydroxybutyrate, and aminomalonic acid, were positively correlated with ESR (26). Our study demonstrated that higher ESR levels in UC patients combined with CDI compared to those without. FC, a non-invasive inflammatory biomarker, are calcium-zinc binding protein complexes belonging to the S100 family, consisting of two hydrophobic, non-covalently linked S100 A8/A9 regions. These proteins account for approximately 60% of the total cytoplasmic protein content in neutrophils, with smaller amounts found in monocytes, macrophages, and epithelial cells. FC is useful for the differential diagnosis of diseases [e.g., UC vs. Crohn's disease (CD), IBD vs. irritable bowel syndrome], assessing inflammatory activity, and predicting short-term relapse. Although FC demonstrates good sensitivity and specificity for diagnosing IBD, its optimal cut-off value can vary depending on factors such as age, diet, lifestyle, disease type, medications, defecation timing, sample storage, and detection methods. The AGA Clinical Practice Guidelines recommend that elevated FC levels may indicate an inflammatory source, and fecal testing for CDI is essential in UC patients with symptoms consistent with the disease and elevated biomarkers (e.g., FC, CRP, fecal lactoferrin) (27). Certainly, FC can also be elevated due to other infections (e.g., *Salmonella*, *Campylobacter*, or certain pathogenic strains of *Escherichia coli*). Children with UC combined with CDI exhibited markedly increased FC levels in our study. Therefore, when there is aggravation of clinical manifestations and elevation of inflammatory markers, clinicians should be alert to opportunistic infections.

The active form of Vit D, 1,25-dihydroxyvitamin D (1,25-(OH)₂-D), plays a critical role in bone formation, calcium and phosphorus metabolism, and cellular growth and differentiation. Increasing evidence suggests that the Vit D/VDR signaling pathway is crucial for maintaining the integrity of the intestinal barrier, modulating immune responses, and regulating the microbiome (28). Tight junctions between intestinal epithelial cells are essential for the intestinal mucosal barrier, controlling permeability. When these tight junctions are impaired (through downregulation and disruption of tight junction proteins), intestinal permeability increases, allowing pathogens to invade and activating a sustained immune response (29). Therefore, the Vit D/VDR system may significantly influence UC development. For instance, Bakke et al. (30) observed reduced VDR expression and impaired Vit D/VDR signaling in UC patients, while a meta-analysis of 987 UC patients and 1,247 controls found significantly lower serum Vit D levels in UC patients (OR = 1.90, 95% CI: 1.38–2.62) (31). Multivariate regression analysis in our study identified hypovitaminosis D as an independent risk factor for CDI in children with UC. β2-defensin, an antimicrobial peptide that inhibits the action of CD toxins and alleviates colitis, is stimulated by plasma 1,25-(OH)₂-D. Based on these mechanisms, Ananthakrishnan et al. investigated the relationship between plasma 1,25-(OH)₂-D and CDI in 188 IBD patients, including 35 combined with CDI. Patients combined with CDI had significantly lower mean plasma 1,25-(OH)₂-D levels compared to those with IBD (20.4 ng/ml vs. 27.1 ng/ml, *p* = 0.002). The risk of CDI decreased by 4% for each 1 ng/ml increase in Vit D levels (OR = 0.96, 95% CI: 0.93–0.99, *p* = 0.046). Furthermore, the risk of CDI was higher in patients with Vit D levels below 20 ng/ml compared to those with levels above 20 ng/ml (OR = 3.1, 95% CI: 1.2–8.3, *p* = 0.02) (32). Our study similarly demonstrated a negative correlation between Vit D levels and the occurrence of children with UC combined with CDI. Consequently, serum Vit D levels were incorporated as a predictive factor in the construction of the children with UC nomogram model. Children with UC, often referred to as the “green tumor”, is associated with reduced Vit D levels due to both non-disease-related factors (e.g., limited sunlight exposure and reduced physical activity) and disease-related factors (e.g., decreased dietary intake, malabsorption, and increased basal metabolism). Children with UC combined with CDI triggers inflammatory response and affects Vit D level.

Environmental risk factors for CDI include antibiotic and proton pump inhibitor (PPI) use, escalation of IBD therapy, prolonged hospitalization, immune dysfunction, and a history of gastrointestinal surgery. The primary defense against CDI is the normal intestinal flora. Antibiotic therapy disrupts the intestinal microbiota, facilitating CDI colonization and toxin production, which damages the cytoskeletal structure of epithelial cells and induces inflammation (33). The risk of CDI is influenced by the type, quantity, and duration of antibiotic use. Antibiotics most frequently associated with CDI include fluoroquinolones, clindamycin, and broad-spectrum penicillins and cephalosporins. A meta-analysis of 14 studies found prior antibiotic exposure was linked to a higher incidence of CDI in children (OR: 2.14; 95% CI: 1.31–3.52) (34). Balram et al. (35) conducted a meta-analysis of 22 studies, showing that antibiotic use within 30 days prior to diagnosis was associated with CDI occurrence in IBD patients (OR: 1.85, 95% CI: 1.36–2.52). Despite being a cornerstone of treatment for inflammatory diseases, frequent antibiotic use has been linked to an increased incidence of CDI in UC patients. In this study, antibiotic use exceeding seven days was found to be a risk factor for CDI combined with children with UC, suggesting that early use of metronidazole or vancomycin, in accordance with guidelines, may reduce the risk of CDI.

The dynamic nomogram prediction model constructed by five routine indicators in this study provides an intuitive and individualized warning tool for clinical practice. To help clinicians risk stratification of low, moderate, and high risk patients, and take active and effective countermeasures, so as to shorten the length of hospital stay, reduce medical costs and improve the efficiency of antibiotic management. Although this study provides a new scheme for the prediction of risk factors for children with UC combined with CDI, it still has some limitations. First, this study is a single-center, small-sample retrospective analytical study, which may suffer from data collection and selection bias. Therefore, it is necessary to carry out a prospective study with multiple centers and large samples in the future, and carry out external validation to further explore other predictive factors and improve the accuracy and stability of the model. Second, the relationship between IBD therapeutic regimens and CDI risk remains incompletely characterized. During clinical data collection, we observed that the pediatric cohort demonstrated low prevalence of conventional risk factors with no statistically significant differences, consequently these variables were excluded from risk factor analysis. Future large-scale clinical trials will incorporate dedicated investigations to elucidate potential associations between treatment modalities and CDI pathogenesis. Third, the genome of CD is mutable, so more virulent strains may emerge. These strains vary in geographic distribution, genetic composition, virulence factors and antibiotic susceptibility. Therefore, this study can only determine whether one is infected with CD or not, and is not applicable to predict specific strain types. Therefore, it is to be explored in further studies in the future. Fourth, substantial heterogeneity exists in reported colectomy rates among IBD patients with concurrent CDI. Future investigations should focus on delineating clinical outcomes in this pediatric subpopulation to optimize therapeutic management strategies.

## Conclusion

The clinical data of children with UC, with or without CDI, were used to develop a dynamic nomogram prediction model by Lasso regression and multifactor logistic regression analyses. Five predictors (PUCAI, ESR, Vit D, FC, and antibiotic use exceeding seven days) were identified. PUCAI, ESR, VitD, FC and antibiotic use exceeding seven days serve as indicators of inflammatory response intensity and immune function status, and may also signal the presence of an inflammatory storm during infection. According to the specific score cut-off value of the model, risk stratification was performed to guide clinical decision-making. The model not only has good discrimination, calibration, clinical utility, but also has good consistency in clinically relevant subgroups (demographic and clinical characteristics). We hope to conduct prospective studies to identify potential predictors, and conduct multi-center external validation to strengthen the extrapolation of the model and improve the efficiency of clinical diagnosis and treatment.

## Data Availability

The raw data supporting the conclusions of this article will be made available by the authors, without undue reservation.
